# Molecular dynamics and predictive toxicity insights into theaflavin as a potential inhibitor of MMP−2 and MMP−9 in breast cancer

**DOI:** 10.1016/j.toxrep.2026.102309

**Published:** 2026-07-05

**Authors:** Iwan Benny Purwowidodo, Farid Sri Lingganingrum, Riska Prasetiawati, Nining Juliani, Taufik Muhammad Fakih, Dhania Novitasari, Ezatul Ezleen Kamarulzaman, Muchtaridi Muchtaridi

**Affiliations:** a Konsep Karnus - PT. Alga Rosan Nusantara, Gading Fajar, Blok A1 No. 21, Buduran, Sidoarjo 61252, Indonesia; b Department of Pharmacy, Faculty of Mathematics and Natural Sciences, Universitas Garut, Jl. Jati, Garut 44151, Indonesia; c Department of Pharmacy, Faculty of Mathematics and Natural Sciences, Universitas Islam Bandung, Jl. Ranggagading, Bandung 40116, Indonesia; d Department of Pharmaceutical Analysis and Medicinal Chemistry, Faculty of Pharmacy, Universitas Padjadjaran, Jl. Raya Bandung-Sumedang, Sumedang 45363, Indonesia; e School of Pharmaceutical Sciences, Universiti Sains Malaysia, USM Health Campus, 11800 Gelugor, Penang, Malaysia; f Research Collaboration Centre for Radiopharmaceuticals Theranostic, National Research and Innovation Agency (BRIN), Jl. Raya Bandung-Sumedang, Sumedang 45363, Indonesia

**Keywords:** Breast cancer, Matrix metalloproteinase−2 (MMP−2), Matrix metalloproteinase−9 (MMP−9), Black tea (*Camellia sinensis*), Molecular simualtions

## Abstract

Breast cancer is one of the most commonly diagnosed malignancies in women and remains a leading cause of cancer-related mortality worldwide. Matrix metalloproteinases (MMPs), particularly Matrix Metalloproteinase−2 (MMP−2) and Matrix Metalloproteinase−9 (MMP−9), play critical roles in cancer invasion, metastasis, and angiogenesis, making them attractive therapeutic targets. Theaflavin, a major polyphenolic compound derived from black tea (*Camellia sinensis*), has demonstrated anticancer properties, notably through modulation of the nuclear factor kappa B (NF−κB) signaling pathway and suppression of MMP expression. This study aims to evaluate the inhibitory potential of theaflavin against MMP−2 and MMP−9 in breast cancer using molecular docking and molecular dynamics (MD) simulations. The ligand and receptor structures were obtained from PubChem and the Protein Data Bank (PDB), respectively. Molecular docking was conducted using AutoDock to predict binding affinities and identify key residue interactions. Thereafter, 200-nanosecond molecular dynamics (MD) simulations were performed using Assisted Model Building with Energy Refinement (AMBER), employing the leap-frog integrator and Linear Constraint Solver (LINCS) constraints to evaluate the dynamic stability of the ligand–protein complexes. Binding free energies were further assessed through Molecular Mechanics Generalized Born Surface Area (MM-GBSA) analysis to estimate thermodynamic contributions to ligand binding. Docking results revealed that theaflavin exhibited a stronger binding affinity toward MMP−9 (−11.91 kcal/mol) compared to MMP−2 (−10.11 kcal/mol), although favorable binding interactions were observed with both targets. In the MMP−9 active site, theaflavin formed stable interactions with key catalytic residues, including HIS226, HIS230, HIS236, and GLU227 within the MMP−9 active site. Theaflavin also demonstrated stable binding within the MMP−2 active site, supporting its potential inhibitory activity against this gelatinase. MD simulations further confirmed the superior stability of the theaflavin–MMP−9 complex, as evidenced by consistent root mean square deviation (RMSD) and root mean square fluctuation (RMSF) fluctuations, compact conformational profiles, and favorable solvent accessibility. MM-GBSA calculations further validated these findings, indicating significant van der Waals and electrostatic contributions to complex stabilization**.** In addition, predictive toxicity analysis was performed to evaluate the safety profile of theaflavin relative to the native ligands. Hence, theaflavin demonstrates inhibitory potential against both MMP−2 and MMP−9, with stronger and more stable interactions observed for MMP−9, suggesting its role as a promising natural compound for breast cancer therapy. These computational findings support the potential of theaflavin as a candidate for further *in vitro* and *in vivo* studies targeting matrix metalloproteinase-driven breast cancer progression.

## 1 Introduction

Breast cancer is among the most frequently diagnosed malignancies in women and continues to be a major cause of cancer-related deaths worldwide. Based on the GLOBOCAN 2022 report, breast cancer ranks as the second most prevalent cancer globally, with 2.3 million new cases accounting for 11.5% of all cancer diagnoses [1]. The mortality toll is equally significant, with 666,103 deaths, representing 6.81% of global cancer-related deaths [2]. In Indonesia, breast cancer is the most commonly diagnosed cancer, with 66,271 reported cases per year, representing 30.1% of all cancer incidence. Furthermore, the mortality burden in Indonesia is concerning, as 22,598 deaths are attributed to breast cancer annually [3], [4]. The persistence of high incidence and mortality rates illustrates the urgent need for novel treatment approaches. Current standard therapies, such as surgery, chemotherapy, and hormonal therapy, while effective, are often inadequate for advanced cases [5]. Moreover, drug resistance and toxicity are persistent clinical problems that limit therapeutic success [6]. This situation highlights the importance of identifying molecular targets and mechanisms for innovative interventions.

Breast cancer progression involves complex biological processes and cellular mechanisms that enhance malignancy [7]. These processes include uncontrolled cell proliferation, sustained angiogenesis, and the evasion of apoptosis [8], [9]. Another critical hallmark is the ability of cancer cells to invade surrounding tissues and metastasize to distant organs. This invasive capacity depends largely on the degradation of the extracellular matrix (ECM) [10]. The breakdown of the ECM not only supports tumor expansion but also facilitates the migration and colonization of secondary sites. Central to this process are matrix metalloproteinases (MMPs), a group of zinc-dependent proteolytic enzymes that regulate ECM remodeling under normal physiological conditions but are commonly dysregulated in cancer [11]. Their overexpression has been linked to aggressive phenotypes, metastatic spread, and tumor angiogenesis [11]. In breast cancer, aberrant MMP activity contributes directly to disease progression and worse clinical outcomes [12]. Understanding the regulatory role of MMPs is therefore essential for designing effective inhibitors. Targeting of MMP activity represents a promising but challenging therapeutic strategy.

Among the broad family of MMPs, MMP−2 and MMP−9 are the most frequently implicated in breast cancer malignancy. These enzymes, known as gelatinases, primarily degrade type IV collagen, a major structural component of the basement membranes [13]. The degradation of basement membranes is a crucial step that enables tumor cells to infiltrate adjacent tissues and establish metastases [14]. Studies have consistently shown that MMP−2 and MMP−9 expression levels are elevated in breast cancer cells compared to normal tissues [15]. This increased expression is strongly associated with advanced clinical stages, lymph node metastasis, and poor overall survival [16]. Hence, these enzymes are regarded as promising therapeutic targets for intervention. However, clinical translation of MMP inhibition has faced major obstacles. MMP−2 is known to be mainly inhibited by TIMP−2, while TIMP−1 primarily inhibits MMP−9 [17]. Several generations of synthetic MMP inhibitors have been designed, yet most have failed in clinical trials due to a lack of selectivity and severe adverse effects. Musculoskeletal toxicity, including joint stiffness and pain, has been commonly reported among patients receiving these inhibitors [18], [19]. Such drawbacks underscore the limitations of synthetic agents and the need to explore alternative therapeutic options. Plant-derived compounds represent one of the most attractive sources in this regard [20].

Natural compounds from medicinal plants are increasingly recognized for their pharmacological benefits in cancer therapy. They often demonstrate multiple biological effects, such as antioxidant, anti-inflammatory, and anticancer activities, with fewer adverse events compared to synthetic drugs [21], [22]. One promising candidate is theaflavin, a major polyphenolic compound found abundantly in black tea (*Camellia sinensis*). Theaflavin is known for its strong antioxidant properties, with an IC_50_ value of 14.5 µg/ml, which effectively mitigates oxidative stress [23]. This is significant because oxidative stress contributes to MMP overexpression through activation of signaling pathways including NF-κB, ERK1/2, and p38 MAPK [24], [25]. *In vitro* findings have demonstrated that theaflavin suppresses MMP−2 and MMP−9 expression by inhibiting NF-κB activation [26]. These findings suggest theaflavin’s potential as an anticancer agent, particularly in targeting invasion and metastasis mechanisms. Nevertheless, the precise molecular interactions between theaflavin and MMP enzymes have not been thoroughly explored. Determining whether theaflavin can directly bind to the active sites of MMP−2 and MMP−9 is a critical step in validating its therapeutic potential. Computational studies offer an effective strategy to address this knowledge gap. Such approaches can guide future experimental validation and drug development.

Molecular docking and molecular dynamics (MD) simulations are powerful *in silico* approaches widely applied in modern drug discovery. Docking analysis predicts potential binding affinities and identifies specific residues involved in ligand–protein interactions [27]. In contrast, MD simulations provide insights into the structural stability and conformational dynamics of complexes under physiological conditions [28]. Together, these computational methods yield valuable mechanistic information prior to experimental validation. They are particularly advantageous when evaluating natural compounds with limited experimental data. In this study, docking and MD simulations were employed to analyze theaflavin’s interactions with MMP−2 and MMP−9. The binding stability was further assessed using root mean square deviation (RMSD), root mean square fluctuation (RMSF), and binding free energy calculations. These analyses provide a robust framework for assessing the therapeutic promise of theaflavin. By focusing on MMP−2 and MMP−9, this study addresses key molecular mediators of breast cancer progression. The findings are expected to contribute to the development of natural compound-based inhibitors for breast cancer therapy. Ultimately, this research highlights the importance of integrating computational tools into the rational design of safer, more effective anticancer agents.

## 2 Materials and methods

### 2.1 Preparation of the ligand structure

**Fig. 1:**
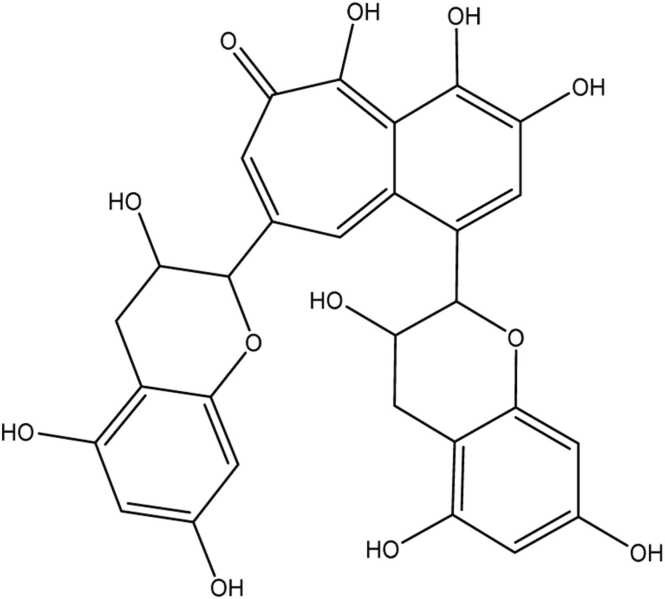
Two-Dimensional Conformation of Theaflavin (PubChem CID: 13543798).

Theaflavin, a polyphenolic compound found in black tea, was selected as the ligand of interest in this study due to its reported anticancer properties. The compound was retrieved from the PubChem database (PubChem CID: 13543798) in three-dimensional (.sdf) format (Fig. 1) [29]. The downloaded structure was initially examined for completeness and correctness to ensure no missing atoms or unusual bond lengths. MarvinSketch software was employed to perform preliminary molecular optimization,correcting any geometrical irregularities [30]. Further optimization was carried out using AutoDock Tools 1.5.7 to prepare the ligand for docking analysis [31]. During this step, torsional degrees of freedom were defined to allow flexibility in ligand conformations. Gasteiger charges were added to the ligand to improve accuracy in electrostatic interaction predictions. The prepared structure was saved in (.pdbqt) format for docking simulations. A careful examination of the energy-minimized structure was conducted to confirm its suitability for further computational studies. The ligand’s molecular geometry was then cross-checked with literature reports to validate consistency. A three-dimensional visualization of theaflavin was generated to illustrate its structural features. This optimized ligand structure served as the starting point for subsequent receptor interaction studies.

### 2.2 Preparation of the MMP−2 and MMP−9 receptor macromolecule

**Fig. 2:**
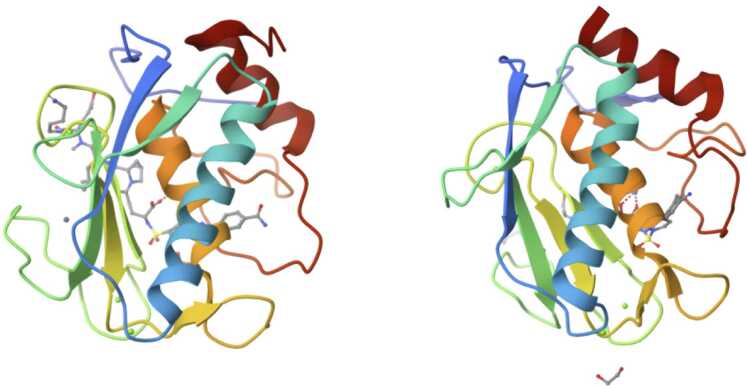
Three-Dimensional Structures of MMP−2 (PDB ID: 8H78, Left) and MMP−9 (PDB ID: 5CUH, Right).

The receptor proteins used in this study were MMP−2 (PDB ID: 8H78) [32] and MMP−9 (PDB ID: 5CUH) [33], obtained from the Protein Data Bank in (.pdb) format (Fig. 2). These structures were carefully selected based on their resolution and structural suitability for molecular docking. Discovery Studio Visualizer was initially employed to examine the crystal structures and remove unwanted molecules, such as water and heteroatoms [34]. Native ligands were separated to prepare the binding pocket for docking with theaflavin. The receptor preparation process continued using AutoDock Tools 1.5.7, where polar hydrogen atoms were added to enhance stability within the binding pocket. Kollman charges were applied to the receptors to account for electrostatic contributions in docking. Similarly, Gasteiger charges were applied to the native ligands to preserve compatibility during validation. After adjustments, all receptor and ligand structures were saved in (.pdbqt) format to ensure compatibility with AutoDock. The receptor binding sites were visually inspected to confirm that active-site residues were preserved. Structural validation was performed by comparing the prepared receptors with their original PDB entries [35]. Three-dimensional representations of both MMP−2 and MMP−9 were generated to aid in visualization and presentation. This receptor preparation ensured reliable docking simulations with theaflavin.

### 2.3 Ligand-receptor molecular docking

Molecular docking was performed using AutoDock Tools 1.5.7 and AutoDock4 to evaluate the binding interactions between theaflavin and the target receptors. The prepared receptors and ligand files in (.pdbqt) format were uploaded into the docking workspace. A grid box was defined around the active site, with X, Y, and Z coordinates selected to encompass the key catalytic residues. This grid ensured that the search space was limited to biologically relevant regions of the receptors. Grid parameter files (.gpf) were generated to define the docking environment. The docking algorithm employed was the Lamarckian Genetic Algorithm (LGA), which efficiently explores conformational space. Docking parameter files (.dpf) were created with the genetic algorithm set to 100 independent runs for accuracy. AutoGrid4 and AutoDock4 were then executed to simulate binding interactions, producing docking log files (.dlg) as output. Binding affinity was expressed in terms of Gibbs free energy (ΔG), with more negative values indicating stronger binding. Amino acid residues interacting with theaflavin were identified and classified as hydrogen bonds, hydrophobic contacts, or electrostatic interactions. Visualization of docking poses and interaction maps was conducted using Discovery Studio Visualizer. These docking results provided initial insights into theaflavin’s ability to interact with MMP−2 and MMP−9.

### 2.4 Ligand-receptor molecular dynamics

Molecular dynamics simulations were performed using AMBER22 to validate docking results and analyze the stability of ligand–receptor complexes [36]. Initially, the docked complexes were merged and processed in Discovery Studio Visualizer for hydrogen atom addition and separation of components. Receptor preparation involved removing extraneous molecules and ensuring appropriate protonation states. Ligand parameterization was performed using the AM1-BCC semi-empirical quantum method within the Antechamber module. Force field parameters were assigned using the ff14SB parameter set for proteins and corresponding parameters for the ligand [37]. The system was solvated with TIP3P water molecules, and counterions were added to neutralize the overall charge. Energy minimization was performed using the steepest descent and conjugate gradient algorithms to eliminate steric clashes. The system was gradually heated to 300 K, followed by equilibration under constant pressure and temperature conditions. A production run of 200 ns was conducted using the pmemd.cuda module for efficient GPU-based simulation. Trajectory files were analyzed using the cpptraj module to calculate RMSD, RMSF, and MM-GBSA binding free energies [38]. These analyses provided comprehensive insights into the stability and interaction dynamics of theaflavin with MMP−2 and MMP−9. The simulation results supported the docking findings and further validated the binding potential of the ligand.

### 2.5 Physicochemical property (druglikeness) prediction

Druglikeness and physicochemical properties of theaflavin were predicted using the SwissADME web server [39]. The compound’s canonical SMILES notation was obtained from PubChem and submitted to the platform. SwissADME evaluates multiple parameters, including lipophilicity, solubility, molecular weight, and hydrogen bond donor/acceptor counts. The results were interpreted according to Lipinski’s Rule of Five, which serves as a guideline for oral bioavailability. Theaflavin’s molecular descriptors were compared against standard thresholds to assess druglikeness. In addition, bioavailability scores and gastrointestinal absorption predictions were analyzed. The presence or absence of PAINS (pan-assay interference compounds) alerts was also checked, as these may indicate promiscuous binding. Results were visualized using the bioavailability radar and BOILED-Egg model provided by SwissADME. These tools allowed rapid assessment of absorption and distribution potential. The analysis further highlighted whether theaflavin could effectively cross biological membranes or the blood–brain barrier. Altogether, these evaluations provided crucial insights into the pharmacological suitability of theaflavin as a drug candidate. The SwissADME predictions complemented docking and MD findings, offering a holistic understanding of the compound’s therapeutic potential.

### 2.6 Pharmacokinetics and toxicity prediction

The toxicity profile of theaflavin was evaluated using the ProTox−3.0 online platform (https://tox.charite.de/protox3/), which employs machine learning models trained on experimental data to predict toxicological endpoints [40]. The canonical SMILES of theaflavin was submitted to the system for in silico screening. ProTox−3.0 generated predictions for several key parameters, including LD_50_ values (oral toxicity), toxicity class classification, and probabilities for hepatotoxicity, cardiotoxicity, immunotoxicity, mutagenicity, and cytotoxicity. These predictions provided insight into the potential safety risks associated with theaflavin. In particular, the LD_50_ estimation and toxicity class assignment offered a preliminary indication of acute toxicity levels *in vivo*. The mutagenicity and carcinogenicity predictions provided additional information on the long-term safety profile. Compared to previously reported toxicity studies of polyphenolic compounds from black tea, theaflavin showed generally consistent patterns, supporting the reliability of the ProTox−3.0 predictions. This computational approach allowed for rapid and cost-effective preliminary screening of toxicological risks without requiring extensive animal testing. The results from ProTox−3.0 provide an important basis for prioritizing theaflavin as a candidate for further experimental safety validation and therapeutic development.

## 3 Results

### 3.1 Ligand-receptor molecular docking

Molecular docking is a computational strategy frequently employed to predict the interactions between small molecules and macromolecular receptors based on their three-dimensional structures. The principal objective of this approach is to identify the most favorable conformation and to calculate the binding free energy that reflects molecular stability [41]. In this study, AutoDock Tools was utilized to perform docking simulations with matrix metalloproteinases MMP−2 and MMP−9 as targets (Table 1). The crystal structure of MMP−2 (PDB ID: 8H78) was obtained at a resolution of 2.4 Å, while MMP−9 (PDB ID: 5CUH) was resolved at 1.83 Å, providing reliable receptor templates. To focus the conformational search on the active binding pockets, grid boxes were carefully defined for both proteins. For MMP−2, the grid box was positioned at X = 28.297, Y = 24.706, and Z = −8.419, with dimensions of 46 × 62 × 40 grid points. For MMP−9, the grid box was set at X = 13.130, Y = 20.881, and Z = −1.944, with dimensions of 40 × 40 × 40 grid points and a spacing of 0.375 Å. The molecular docking analysis revealed that the native ligand of MMP−2 had a Gibbs free energy (ΔG) of −15.95 kcal/mol. In comparison, theaflavin docked into MMP−2 with a ΔG of −10.11 kcal/mol, indicating weaker binding affinity. This finding suggests that the native ligand forms a more stable complex with MMP−2 than theaflavin. The difference in binding energy highlights the variation in ligand compatibility and affinity between different metalloproteinases.

**Table 1:** Molecular docking results of theaflavin and native ligands with MMP−2 (8H78) and MMP−9 (5CUH).

In contrast to MMP−2, the results for MMP−9 demonstrated a stronger binding potential for theaflavin compared to its native ligand. Theaflavin exhibited a binding free energy of −11.91 kcal/mol, which was slightly more negative than the ΔG of the native ligand at −11.80 kcal/mol. This subtle but notable difference indicates that theaflavin may establish a more stable complex with MMP−9, suggesting inhibitory potential. Detailed analysis of amino acid interactions showed that theaflavin reproduced hydrogen bonding patterns similar to those of the native ligand in MMP−2, especially with ALA86 and LEU83. Within the MMP−9 binding pocket, however, theaflavin interacted through hydrogen bonds with HIS226, HIS230, HIS236, and GLU227. These histidine residues play essential roles in coordinating Zn²⁺ ions and supporting catalytic activity [42], whereas GLU227 is critical for ligand stabilization [43]. Furthermore, a hydrophobic π–π stacking interaction was observed with HIS226, which could contribute to enhanced selectivity of binding. Such interactions indicate that theaflavin not only fits within the active site of MMP−9 but also engages residues essential for enzymatic activity. This combination of hydrogen bonding and aromatic stacking underscores its potential as a selective inhibitor [44]. Taken together, these molecular docking outcomes suggest that theaflavin displays stronger inhibitory tendencies against MMP−9 than against MMP−2. These computational findings provide a rational basis for further validation through molecular dynamics simulations and experimental studies. This disparity may stem from substrate preferences, resulting in distinct cellular functions. Notably, MMP−2 plays a role in tumor angiogenesis, while MMP−9 facilitates tumor cell invasion and metastasis [45].

### 3.2 Stability evaluation of theaflavin–MMP complexes

**Fig. 3:**
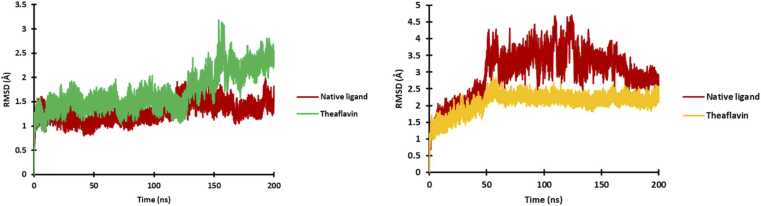
RMSD Plots of the Native Ligand and Theaflavin Bound to MMP−2 (Left) and MMP−9 (Right) Receptors.

Molecular dynamics is widely recognized as a powerful computational method to evaluate the temporal stability of protein–ligand complexes under simulated physiological conditions. By replicating solvent, ion, and temperature environments, the technique provides a more realistic representation of biological interactions than static docking alone [56]. In this study, simulations were carried out for 200 ns using AMBER, and trajectory analysis was performed to examine the structural stability of complexes involving theaflavin and matrix metalloproteinases (MMP−2 and MMP−9). One of the main indicators assessed was the Root Mean Square Deviation (RMSD), which measures the extent of atomic displacement relative to the initial structure. A stable RMSD profile typically indicates that the system has equilibrated and maintains consistent conformations (Fig. 3). For the MMP−2 complex, theaflavin exhibited an average RMSD value of 1.69 Å, with its peak fluctuation reaching 3.17 Å. In comparison, the native ligand demonstrated lower fluctuations, with an average RMSD of 1.29 Å and a maximum of 1.98 Å. These findings suggest that the native ligand had better conformational stability within the MMP−2 binding site than theaflavin. Such stability could imply a stronger or more optimized binding environment for the native ligand compared to the test compound. Nevertheless, theaflavin still maintained an RMSD within the acceptable range for stable ligand–receptor complexes, which typically falls between 2–5 Å. Moreover, the analysis indicates that while theaflavin interacts effectively with MMP−2, its stability is comparatively lower than that of the native ligand.

In contrast, the RMSD analysis of the MMP−9 receptor complex showed a different trend, reflecting unique binding characteristics. Theaflavin exhibited an average RMSD of 2.09 Å, with its maximum fluctuation reaching 2.85 Å throughout the simulation. Interestingly, the native ligand bound to MMP−9 displayed higher instability, with an average RMSD of 2.97 Å and a maximum deviation of 4.71 Å. This suggests that theaflavin was able to maintain a more consistent binding orientation within the MMP−9 active site compared to the native ligand. The lower RMSD values of theaflavin indicate a favorable interaction profile and potentially greater structural stability within the receptor. Such stability can be correlated with enhanced binding efficiency and diminished conformational drift during the simulation duration. These results are significant because they indicate that theaflavin may act as a more effective inhibitor of MMP−9 than the native ligand. Stable RMSD behavior suggests that the ligand is capable of adapting well to the dynamic fluctuations of the protein. The consistency in its trajectory further strengthens the hypothesis that theaflavin has therapeutic potential against MMP−9. Altogether, while theaflavin showed reduced stability with MMP−2, it demonstrated enhanced stability with MMP−9, highlighting its receptor-specific interaction properties.

### 3.3 Flexibility analysis of theaflavin–MMP complexes

**Fig. 4:**
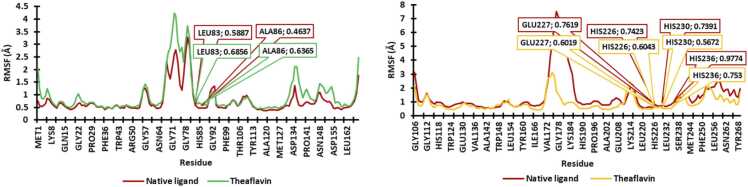
RMSF Plots of the Native Ligand and Theaflavin Bound to MMP−2 (Left) and MMP−9 (Right) Receptors.

Root Mean Square Fluctuation (RMSF) is a crucial parameter in molecular dynamics simulations, used to assess the flexibility of individual amino acid residues over the simulation time scale. Low RMSF values typically indicate residues with limited mobility, suggesting they contribute to stable ligand–receptor interactions at the binding site [46]. Conversely, residues with higher fluctuations are generally considered less stable and more mobile, reflecting structural flexibility. In this study, RMSF analysis was performed on the MMP−2 receptor complexed with both the native ligand and the test compound theaflavin (Fig. 4). The native ligand displayed fluctuations of 0.58 Å at residue LEU83 and 0.46 Å at ALA86, indicating highly stable interactions at these key residues. Theaflavin, in comparison, exhibited slightly higher fluctuations of 0.68 Å at LEU83 and 0.60 Å at ALA86, indicating somewhat greater flexibility. Both ligands, however, demonstrated fluctuations below the 1.4 Å threshold, which is commonly accepted as the benchmark for stable interactions. Despite this stability, the average RMSF value of theaflavin across all residues was 0.96 Å, compared to 0.75 Å for the native ligand. This difference suggests that while both ligands bind effectively at the active site, theaflavin may introduce slightly more dynamic flexibility. Such flexibility could potentially reduce the overall stability of the theaflavin–MMP−2 complex when compared to the native ligand. Nevertheless, theaflavin maintained acceptable stability throughout the simulation period, supporting the relevance of its binding profile.

For the MMP−9 receptor, the RMSF analysis revealed a different trend in terms of ligand stability and residue flexibility. The native ligand exhibited fluctuations of 0.74 Å at HIS226, 0.73 Å at HIS230, 0.97 Å at HIS236, and 0.76 Å at GLU227, all within the stable range. In contrast, theaflavin demonstrated lower fluctuation values at the same residues, with 0.60 Å at HIS226, 0.56 Å at HIS230, 0.75 Å at HIS236, and 0.60 Å at GLU227. These results clearly suggest that theaflavin binds more tightly and stably to the catalytic residues of MMP−9 compared to the native ligand. The lower RMSF values for theaflavin highlight its ability to reduce residue-level flexibility at the binding site, thereby supporting the formation of a more stable interaction. Additionally, the average RMSF for theaflavin complex was 0.91 Å, which was notably lower than that of the native ligand average of 1.40 Å. This suggests that the overall structural fluctuations of MMP−9 were dampened in the presence of theaflavin. Reduced flexibility of critical catalytic residues is particularly important, as it may limit enzymatic activity and thus enhance inhibitory potential. The findings imply that theaflavin has a strong capacity to stabilize the dynamic conformation of MMP−9. Moreover, the consistency of these RMSF results further strengthens the docking predictions that indicated favorable binding of theaflavin. Taken together, although theaflavin showed slightly less stability with MMP−2, it demonstrated enhanced stability with MMP−9, indicating its receptor-specific interaction advantage.

### 3.4 MM-GBSA-based assessment of ligand–receptor stability

MM-GBSA (Molecular Mechanics-Generalized Born Surface Area) is a widely used method to calculate the binding free energy of protein–ligand complexes by incorporating multiple energetic contributions. This approach evaluates van der Waals interactions, electrostatic energy, electrostatic contributions to solvation free energy, and non-polar solvation free energy in a cumulative manner. Unlike docking scores, which provide an initial estimate of binding affinity, MM-GBSA integrates data from molecular dynamics simulations, offering a more reliable description of ligand–receptor interactions. A lower ΔG value indicates a more stable and favorable binding, as the system requires less free energy to maintain the interaction [57]. The method has become a gold standard for assessing post-docking stability in computational drug design. In the context of this study, MM-GBSA was performed to differentiate the stability of theaflavin compared to native ligands bound to MMP−2 and MMP−9 receptors (Table 2). The importance of this analysis lies in its ability to highlight conformational behavior beyond static docking poses. Furthermore, the interpretation of free energy values helps determine whether theaflavin demonstrates meaningful inhibitory potential. Hence, MM-GBSA acts as a bridge between molecular docking predictions and experimental feasibility. This integrated energy assessment strengthens the conclusions drawn from other simulation parameters such as RMSD and RMSF.

**Table 2:** MM-GBSA values of native ligands and theaflavin with MMP−2 and MMP−9.

**Energy Component (kcal/mol)**	**MMP−2**		**MMP−9**	
	Native L2U (kJ/mol)	Theaflavin (kJ/mol)	Native LTQ (kJ/mol)	Theaflavin (kJ/mol)
Van der Waals Interaction (VdW)	−86.9633	−16.9033	−40.498	−30.4393
Electrostatic Energy (EEL)	30.8387	127.5309	533.1618	405.7810
Electrostatic Contribution to Solvation Free Energy (EGB)	−9.3666	−115.5333	−480.4355	−379.1342
Non-polar Contribution to Solvation Free Energy (ESURF)	−11.0781	−2.2772	−5.2491	−3.8541
∆Ggas (VdW+EEL)	−56.1246	110.6277	492.6671	375.3417
∆Gsolv (EGB + ESURF)	−20.4447	−117.8105	−485.6846	−382.9883
∆GTOTAL (VdW + EEL + EGB + ESURF)	−76.5694	−7.1828	6.9825	−7.6466

Based on the MM-GBSA analysis, the MMP−2 complex with its native ligand produced a total ΔG value of –76.56 kcal/mol, significantly more negative than theaflavin’s value of –7.18 kcal/mol. This strongly indicates that the native ligand has superior binding affinity and stability within MMP−2 compared to theaflavin. Thus, theaflavin is unlikely to act as an effective inhibitor of MMP−2 based on this result. In contrast, analysis of the MMP−9 receptor complex showed a different trend, in which theaflavin demonstrated a ΔG of –7.64 kcal/mol, more negative than the native ligand’s positive ΔG value of 6.98 kcal/mol. A more negative ΔG represents stronger binding affinity, while a positive ΔG implies unfavorable binding interactions. This suggests that theaflavin can bind more stably and tightly to MMP−9 than the native ligand. These results align with the docking predictions, further validating the preferential binding of theaflavin to MMP−9. The role of electrostatic and van der Waals contributions appears to dominate its binding profile, supporting its inhibitory potential. Furthermore, these findings suggest that theaflavin is a promising candidate for MMP−9 inhibition but not for MMP−2. Therefore, future research may prioritize the validation of MMP−9 inhibition by theaflavin through *in vitro* or *in vivo* studies.

### 3.5 Prediction of physicochemical and druglikeness properties

The physicochemical properties of the native ligands and theaflavin reveal clear differences that influence their potential drug-likeness (Table 3). The native ligand L2U exhibited a very high molecular weight of 916.01 g/mol, with 64 heavy atoms and 30 rotatable bonds, which clearly violate Lipinski’s Rule of Five. Similarly, theaflavin also showed a relatively high molecular weight of 564.49 g/mol and 41 heavy atoms, accompanied by 23 aromatic atoms, which exceed the optimal range for oral bioavailability. In contrast, the native LTQ demonstrated a smaller molecular weight of 411.50 g/mol, comprising only 27 heavy atoms and 5 rotatable bonds, which aligns more closely with favorable drug-likeness criteria. In terms of solubility, L2U was categorized as very soluble, with an ESOL-predicted solubility of 3.46e + 01 mg/ml, whereas theaflavin was moderately soluble, with a value of 5.39e−02 mg/ml, indicating limited aqueous solubility. LTQ, however, was classified as soluble, with a predicted solubility of 1.17e + 00 mg/ml. The predicted topological polar surface area (TPSA) also highlighted differences, with L2U having the highest TPSA of 369.27 Å², LTQ at 150.66 Å², and theaflavin at 217.60 Å². A higher TPSA generally correlates with poor cell membrane permeability, which could limit oral absorption. Log P values also varied, with theaflavin showing a consensus Log P of 0.90, suggesting moderate lipophilicity compared to L2U (–1.37) and LTQ (0.14). These results indicate that while LTQ aligns with most drug-likeness criteria, theaflavin and L2U show multiple violations that could affect their pharmacokinetic properties.

**Table 3:** Physicochemical properties of the selected compounds obtained from in silico prediction.

**Parameter**	**Native L2U**	**Native LTQ**	**Theaflavin**
Formula	C_41_H_57_N_9_O_13_S	C_16_H_21_N_5_O_4_S2	C_29_H_24_O_12_
Molecular weight	916.01 g/mol	411.50 g/mol	564.49 g/mol
Num. heavy atoms	64	27	41
Num. arom. heavy atoms	12	6	23
Fraction Csp3	0.49	0.50	0.21
Num. rotatable bonds	30	5	2
Num. H-bond acceptors	15	7	12
Num. H-bond donors	9	2	9
Molar Refractivity	232.78	111.79	143.98
TPSA	369.27 Å²	150.66 Å²	217.60 Å²
Log P (iLOGP)	1.31	1.11	1.53
Log P (XLOGP3)	−3.58	0.51	0.63
Log P (WLOGP)	−0.02	0.05	1.56
Log P (MLOGP)	−4.45	0.22	−0.79
Log P (SILICOS-IT)	−0.11	−1.17	1.56
Consensus Log P	−1.37	0.14	0.90
Log S (ESOL)	−1.42	−2.55	−4.02
Solubility (ESOL)	3.46e + 01 mg/ml; 3.78e−02 mol/l	1.17e + 00 mg/ml; 2.84e−03 mol/l	5.39e−02 mg/ml; 9.55e−05 mol/l
Class (ESOL)	Very soluble	Soluble	Moderately soluble
Log S (Ali)	−3.59	−3.24	−4.77
Solubility (Ali)	2.35e−01 mg/ml; 2.57e−04 mol/l	2.34e−01 mg/ml; 5.70e−04 mol/l	9.49e−03 mg/ml; 1.68e−05 mol/l
Class (Ali)	Soluble	Soluble	Moderately soluble
Log S (SILICOS-IT)	−5.88	−1.96	−4.22
Solubility (SILICOS-IT)	1.20e−03 mg/ml; 1.31e−06 mol/l	4.49e + 00 mg/ml; 1.09e−02 mol/l	3.39e−02 mg/ml; 6.01e−05 mol/l
Class (SILICOS-IT)	Moderately soluble	Soluble	Moderately soluble
GI absorption	Low	Low	Low
BBB permeant	No	No	No
P-gp substrate	Yes	Yes	No
CYP1A2 inhibitor	No	No	No
CYP2C19 inhibitor	No	No	No
CYP2C9 inhibitor	No	No	No
CYP2D6 inhibitor	No	No	No
CYP3A4 inhibitor	Yes	No	No
Log Kp (skin permeation)	−14.43 cm/s	−8.45 cm/s	−9.30 cm/s
Lipinski	No; 3 violations: MW> 500, NorO> 10, NHorOH> 5	Yes; 0 violation	No; 3 violations: MW> 500, NorO> 10, NHorOH> 5
Ghose	No; 3 violations: MW> 480, MR> 130, #atoms> 70	Yes	No; 2 violations: MW> 480, MR> 130
Veber	No; 2 violations: Rotors> 10, TPSA> 140	No; 1 violation: TPSA> 140	No; 1 violation: TPSA> 140
Egan	No; 1 violation: TPSA> 131.6	No; 1 violation: TPSA> 131.6	No; 1 violation: TPSA> 131.6
Muegge	No; 6 violations: MW> 600, XLOGP3 < −2, TPSA> 150, Rotors> 15, H-acc> 10, H-don> 5	No; 1 violation: TPSA> 150	No; 3 violations: TPSA> 150, H-acc> 10, H-don> 5
Bioavailability Score	0.11	0.55	0.17
PAINS	0 alert	0 alert	1 alert: catechol_A
Brenk	0 alert	2 alerts: hydroxamic_acid, oxygen-nitrogen_single_bond	1 alert: catechol
Leadlikeness	No; 2 violations: MW> 350, Rotors> 7	No; 1 violation: MW> 350	No; 1 violation: MW> 350
Synthetic accessibility	7.03	4.19	5.11

The bioavailability and ADMET predictions further highlight the limitations of theaflavin compared to native ligands. The bioavailability score of theaflavin was only 0.17, significantly lower than that of LTQ at 0.55, while L2U scored even lower at 0.11. Both L2U and theaflavin violated three Lipinski criteria, primarily due to their high molecular weight, excessive hydrogen bond donors/acceptors, and large TPSA values, indicating reduced potential for oral bioavailability. LTQ, on the other hand, passed Lipinski without any violations, supporting its suitability for further optimization. None of the compounds were predicted to cross the blood–brain barrier (BBB), suggesting limited central nervous system exposure. Theaflavin showed no P-gp substrate activity, unlike both native ligands, which may confer a pharmacokinetic advantage in terms of efflux resistance. However, toxicity alerts were present, with theaflavin flagged for catechol-related PAINS and Brenk alerts, which may suggest potential assay interference. L2U showed no PAINS alerts but had higher synthetic accessibility scores, indicating greater complexity in synthesis compared to LTQ. Additionally, LTQ emerged as the most promising candidate with favorable physicochemical, solubility, and drug-likeness properties, while theaflavin demonstrated moderate potential due to limitations in solubility, permeability, and bioavailability. These insights highlight that while theaflavin interacts well at the molecular level, its physicochemical properties may hinder its development as an orally active therapeutic agent without structural modifications.

### 3.6 Pharmacokinetics and toxicity prediction

The in silico toxicity prediction provides important insights into the potential safety profiles of theaflavin compared with the native ligands (Table 4). Native L2U showed limited toxicity, being active only for respiratory toxicity and clinical toxicity, while remaining inactive for the majority of other endpoints. Similarly, native LTQ displayed a toxicity profile characterized by activity in respiratory toxicity and clinical toxicity, while remaining inactive for hepatotoxicity, neurotoxicity, nephrotoxicity, cardiotoxicity, mutagenicity, and ecotoxicity. In contrast, theaflavin demonstrated activity in several key categories, including respiratory toxicity, nephrotoxicity, immunotoxicity, blood–brain barrier (BBB) penetration, clinical toxicity, and nutritional toxicity. This broader spectrum suggests that although theaflavin may have therapeutic potential, its safety risks must be carefully considered. Importantly, theaflavin was predicted to be inactive for hepatotoxicity, neurotoxicity, cytotoxicity, mutagenicity, carcinogenicity, and ecotoxicity, indicating the absence of several major toxicity liabilities commonly associated with drug candidates. The active respiratory toxicity prediction indicates a potential association with respiratory-related toxicity endpoints; however, the specific toxicological manifestations cannot be determined from this computational prediction alone and require experimental validation. Likewise, the predicted nephrotoxicity warrants careful investigation in future studies, as renal safety is an important consideration during drug development. The active immunotoxicity endpoint highlights the possibility that theaflavin could modulate immune responses, either beneficially or detrimentally, depending on the dosage and context. Its activity at the blood-brain barrier (BBB) suggests that theaflavin has the potential to cross into the central nervous system, although BBB activity does not necessarily indicate neurotoxicity and should be interpreted cautiously. The clinical toxicity and nutritional toxicity endpoints represent broad predictive categories within the toxicity model and do not directly correspond to specific pathological conditions. These categories are intended to provide a general indication of potential toxicological concern and should not be interpreted as evidence of a particular disease state, organ-specific injury, or metabolic disorder without supporting experimental data. Therefore, these predictions should be interpreted as indicators of potential biological or physiological effects rather than definitive adverse outcomes. Thus, the toxicity profile of theaflavin reveals both strengths and weaknesses, necessitating a balanced evaluation for therapeutic development.

**Table 4:** Predicted toxicity endpoints of theaflavin based on in silico analysis.

**Target**	**Native L2U**	**Native LTQ**	**Theaflavin**
Hepatotoxicity	Inactive	Inactive	Inactive
Neurotoxicity	Inactive	Inactive	Inactive
Nephrotoxicity	Inactive	Inactive	Active
Respiratory toxicity	Active	Active	Active
Cardiotoxicity	Inactive	Inactive	Inactive
Carcinogenicity	Inactive	Inactive	Inactive
Immunotoxicity	Inactive	Inactive	Active
Mutagenicity	Inactive	Inactive	Inactive
Cytotoxicity	Inactive	Inactive	Inactive
BBB-barrier	Inactive	Inactive	Active
Ecotoxicity	Inactive	Inactive	Inactive
Clinical toxicity	Active	Active	Active
Nutritional toxicity	Inactive	Inactive	Active

Comparing the three ligands, theaflavin appears to strike a balance between safety and risk when contrasted with native ligands. While Native L2U and Native LTQ display a more restricted range of active toxicity endpoints, theaflavin’s activity across several categories suggests that its polyphenolic structure may interact with multiple biological targets, thereby broadening its toxicological footprint. The absence of hepatotoxicity and neurotoxicity in theaflavin is a positive indication, as these toxicities are often major limiting factors in drug development. Furthermore, the inactivity toward carcinogenicity and mutagenicity implies that theaflavin is unlikely to induce DNA damage or tumorigenesis, a favorable property for anticancer applications. However, its predicted activity in respiratory toxicity, nephrotoxicity, and immunotoxicity highlights the need for additional *in vitro* and *in vivo* validation to confirm these risks. The BBB penetration activity of theaflavin is noteworthy, as it may open opportunities for its development in brain-targeted cancer therapy, although its toxicological significance cannot be determined solely from this prediction. Similarly, the nutritional toxicity endpoint should be interpreted cautiously because it represents a broad predictive category within the toxicity model. This endpoint may reflect potential interactions with nutritional or physiological processes; however, it does not directly indicate a specific adverse biological outcome. Therefore, the biological significance of this prediction cannot be established without further experimental investigation. By comparison, Native LTQ and Native L2U exhibited fewer active toxicity endpoints than theaflavin, with both compounds showing activity only in respiratory toxicity and clinical toxicity. Because the toxicity model classifies endpoints as active or inactive, these predictions should not be interpreted as indicators of toxicity severity without additional quantitative evidence. Altogether, theaflavin exhibits a complex toxicity profile, balancing therapeutic promise against safety risks, making it an attractive but cautious candidate for further breast cancer therapy research. *In vivo* evaluation of theaflavins has not been reported, rendering this predicted toxicity a preliminary source of information regarding their subsequent *in vivo* toxicity assessment. However, Schuck et al. (2008) has demonstrated that theaflavins can induce cellular damage under specific conditions, including the production of reactive oxygen species that can trigger apoptosis in certain cell types [47].

## 4 Discussions

The findings of this study highlight that theaflavin exhibits stronger interaction stability with MMP−9 compared to MMP−2, which is consistent with the concept that polyphenolic compounds selectively modulate matrix metalloproteinases involved in cancer invasion. Previous studies have demonstrated that black tea polyphenols exert chemopreventive effects in breast cancer through both antioxidant and pro-apoptotic mechanisms, which strengthens the biological plausibility of our results. For example, Kaur et al. (2007) showed that black tea theaflavins significantly reduced mammary tumor size in a transgenic mouse model by promoting apoptosis and reducing oxidative DNA adducts [48]. This aligns with our observation that theaflavin maintains stable interactions with residues critical for the catalytic activity of MMP−9, suggesting a mechanistic pathway for its anticancer activity. Furthermore, the RMSD and MM-GBSA analyses in our study support stable receptor–ligand binding that mirrors experimental observations of enhanced apoptosis in cancer cells treated with tea polyphenols. Although both MMP−2 and MMP−9 exhibit gelatinase activity, they differ in their specific targets and expression patterns. MMP−9 primarily degrades components of the extracellular matrix (ECM) to modulate tumor invasion and is constitutively expressed only in neutrophils, becoming active upon stimulation. In contrast, MMP−2 not only targets gelatin but also type I and IV collagen, and is constitutively expressed in various cell types, where it plays a key role in angiogenesis by facilitating new blood vessel formation [49]. The favorable docking score obtained for theaflavin against MMP−2 (−10.11 kcal/mol) suggests that this compound may also contribute to the suppression of angiogenesis-related processes mediated by MMP−2. Moreover, the stability observed during molecular dynamics simulations indicates that theaflavin can maintain productive interactions within the MMP−2 binding pocket, supporting its potential as a dual MMP−2/MMP−9 inhibitor. Notably, the selectivity toward MMP−9, a protein highly implicated in breast cancer metastasis, provides a rationale for prioritizing this enzyme as a therapeutic target. These outcomes suggest that computational predictions are not only theoretically sound but also corroborated by *in vivo* chemoprevention data. The convergence of computational and biological evidence strongly supports theaflavin as a candidate compound. Moreover, this reinforces the notion that black tea polyphenols should not be overlooked despite the dominance of green tea catechins in the literature. Taken together, our findings expand the scope of natural compounds considered for MMP inhibition in breast cancer therapy.

In addition to the present results, comparative studies have emphasized that natural compounds often provide more favorable safety profiles compared to synthetic MMP inhibitors. Polyphenols from black tea significantly inhibited breast cancer cell invasion by downregulating MMP expression without the severe side effects typically observed with synthetic agents [26]. This complements our findings, in which theaflavin exhibited superior stability with MMP−9 compared to its native ligand, suggesting that such compounds could serve as effective yet safer alternatives. This observation was further supported by predictive toxicity analysis, which indicated that theaflavin exhibited no predicted hepatotoxicity, neurotoxicity, cardiotoxicity, carcinogenicity, mutagenicity, cytotoxicity, or ecotoxicity. However, potential respiratory toxicity, nephrotoxicity, immunotoxicity, and blood–brain barrier activity were identified, suggesting that additional toxicological investigations are required to validate its safety profile. Nevertheless, theaflavin also displayed favorable binding affinity and maintained structural stability in complex with MMP−2, indicating that its inhibitory activity may extend beyond metastasis-associated pathways to include angiogenesis-related mechanisms regulated by MMP−2. Unlike clinical trials of synthetic MMP inhibitors, which failed due to musculoskeletal complications, the use of dietary polyphenols such as theaflavin offers a more tolerable approach to targeting metastasis-related pathways [50], [51]. Furthermore, previous mechanistic studies have shown that NF−κB and MAPK pathways are central mediators of MMP overexpression, both of which are suppressed by theaflavin and related compounds. The structural stability observed in molecular dynamics simulations may provide the basis for such regulatory effects at the molecular level. These computational insights support the idea that theaflavin directly interacts with catalytic residues, thereby inhibiting enzymatic function while also modulating upstream signaling. This dual mechanism could explain the consistent findings across *in silico*, *in vitro*, and *in vivo* studies. Thus, theaflavin emerges as a promising agent that bridges computational predictions with biological relevance. In addition to theaflavins, other flavonoids such as luteolin, [52], hispidulin [53], and wogonin [54], also demonstrate substantial inhibitory activity against MMP−2 and MMP−9. Luteolin specifically decreased the expression of MMP−2 and MMP−9 through the PI3K/AKT signaling pathway [52]. Conversely, wagonin exhibited specific inhibition of MMP−9 activity, thereby reducing the invasion and migration of MHCC97L and PLC/PRF/5 hepatocarcinoma cells [54].

Finally, epidemiological and experimental data provide further context for the significance of tea-derived polyphenols in breast cancer prevention. Way et al. (2004) reported that consumption of tea extracts, particularly theaflavins, reduced mammary tumor burden in rodent models through the reduction of oxidative stress and the induction of apoptosis [55]. This observation resonates with our MM-GBSA results, in which theaflavin demonstrated stronger binding affinity with MMP−9 than its native ligand, highlighting the importance of stable interactions in driving functional outcomes. Our findings also suggest that theaflavin’s effectiveness may not be restricted to direct enzyme inhibition but may also extend to the modulation of oxidative and apoptotic pathways. Such a multi-targeted effect represents a crucial advantage over single-target synthetic inhibitors. Moreover, the consistency of outcomes across molecular docking, dynamics, and binding energy decomposition underscores the reliability of in silico methods for predicting biologically meaningful interactions. While the pharmacokinetic limitations of theaflavin remain a challenge, these limitations can potentially be addressed through novel drug delivery approaches such as nanoformulation. Taken together, this study contributes to the growing body of evidence supporting the therapeutic potential of black tea polyphenols in breast cancer, particularly in targeting gelatinases involved in tumor progression, including MMP−2 and MMP−9, with a stronger interaction profile observed for MMP−9. The results support the need for further preclinical validation to substantiate these computational findings and explore their translational potential.

## 5 Conclusions

In conclusion, this study demonstrated that theaflavin has strong potential as a natural inhibitor of both MMP−2 and MMP−9 in breast cancer. Molecular docking revealed that theaflavin exhibited favorable binding affinity toward MMP−9 (ΔG = –11.91 kcal/mol), which was more stable compared to its binding with MMP−2 (ΔG = –10.11 kcal/mol), indicating effective interactions with both gelatinases while showing a preference for MMP−9. Theaflavin showed consistent interactions with key catalytic residues such as HIS226, HIS230, HIS236, and GLU227. Molecular dynamics simulations further confirmed theaflavin’s stability within the binding pockets of both targets, with the MMP−9 complex exhibiting superior stability, as reflected by an average RMSD of 2.09 Å and maximum deviation of 2.85 Å, compared to the native ligand’s higher average RMSD of 2.97 Å and maximum of 4.71 Å. RMSF analysis also supported these findings, as theaflavin exhibited lower residue fluctuations at active site positions (0.60–0.75 Å) compared to the native ligand (0.73–0.97 Å). Additionally, MM-GBSA calculations indicated that theaflavin formed a stable complex with MMP−9 (ΔG = –7.64 kcal/mol), while the native ligand exhibited a weaker binding affinity and unfavorable binding energy (+6.98 kcal/mol). The overall computational results suggest that theaflavin possesses inhibitory potential against both MMP−2 and MMP−9, which are important mediators of breast cancer invasion and metastasis. These computational findings are consistent with prior experimental reports on black tea polyphenols, which demonstrated suppression of MMP activity and cancer progression. Furthermore, predictive toxicity analysis revealed that theaflavin exhibited no predicted hepatotoxicity, neurotoxicity, cardiotoxicity, carcinogenicity, mutagenicity, cytotoxicity, or ecotoxicity, although potential respiratory toxicity, nephrotoxicity, immunotoxicity, and blood–brain barrier activity were identified, highlighting the need for further safety evaluation in future studies. Collectively, this study highlights theaflavin’s potential as a promising lead compound for targeting MMP−2 and MMP−9, with a stronger and more stable interaction profile toward MMP−9, for breast cancer therapy, warranting further experimental validation through *in vitro* and *in vivo* studies.

## Disclosure

The authors declare there is no conflict of interest.

## Funding

This research did not receive any specific grant from funding agencies in the public, commercial, or not-for-profit sectors.

## CRediT authorship contribution statement

**Farid Sri Lingganingrum:** Visualization, Validation, Software, Resources, Methodology, Investigation, Formal analysis, Data curation. **Iwan Benny Purwowidodo:** Writing – review & editing, Writing – original draft, Visualization, Validation, Supervision, Software, Resources, Project administration, Methodology, Investigation, Funding acquisition, Formal analysis, Data curation, Conceptualization. **Dhania Novitasari:** Writing – review & editing, Visualization, Validation, Supervision, Software, Resources, Methodology, Investigation, Formal analysis, Data curation, Conceptualization. **Taufik Muhammad Fakih:** Writing – review & editing, Writing – original draft, Visualization, Validation, Supervision, Software, Resources, Project administration, Methodology, Investigation, Funding acquisition, Formal analysis, Data curation, Conceptualization. **Muchtaridi Muchtaridi:** Writing – review & editing, Writing – original draft, Visualization, Validation, Supervision, Software, Resources, Project administration, Methodology, Investigation, Funding acquisition, Formal analysis, Data curation, Conceptualization. **Ezatul Ezleen Kamarulzaman:** Writing – review & editing, Visualization, Validation, Supervision, Software, Resources, Methodology, Investigation, Formal analysis, Data curation, Conceptualization. **Nining Juliani:** Writing – original draft, Visualization, Validation, Supervision, Software, Resources, Methodology, Investigation, Formal analysis, Data curation, Conceptualization. **Riska Prasetiawati:** Writing – original draft, Visualization, Validation, Supervision, Software, Methodology, Investigation, Formal analysis, Data curation, Conceptualization.

## Declaration of Competing Interest

The authors declare that they have no known competing financial interests or personal relationships that could have appeared to influence the work reported in this paper.

## Data Availability

The authors do not have permission to share data.
